# Demonstratives in Spatial Language and Social Interaction: An Interdisciplinary Review

**DOI:** 10.3389/fpsyg.2020.555265

**Published:** 2020-11-25

**Authors:** Holger Diessel, Kenny R. Coventry

**Affiliations:** ^1^Department of English, Friedrich-Schiller-Universität Jena, Jena, Germany; ^2^School of Psychology, University of East Anglia, Norwich, United Kingdom

**Keywords:** demonstrative, deixis, joint attention, peripersonal action space, language acquisition, language universals, spatial cognition, embodied cognition

## Abstract

This paper offers a review of research on demonstratives from an interdisciplinary perspective. In particular, we consider the role of demonstratives in current research on language universals, language evolution, language acquisition, multimodal communication, signed language, language and perception, language in interaction, spatial imagery, and discourse processing. Traditionally, demonstratives are analyzed as a particular class of spatial deictics. Yet, a number of recent studies have argued that space is largely irrelevant to deixis and that demonstratives are primarily used for social and interactive purposes. Synthesizing findings in the literature, we conclude that demonstratives are a very special class of linguistic items that are foundational to both spatial and social aspects of language and cognition.

## Introduction

The term “demonstrative” refers to a small class of expressions that are commonly divided into two basic types: nominal demonstratives such as English *this* and *that* and adverbial demonstratives such as *here* and *there* (Dixon, 2003). The two types of demonstratives are closely related. They usually include the same deictic roots (Diessel, 1999) and are defined by two basic concepts of language and cognition, i.e., joint attention and deixis (Levinson, 2004; Diessel, 2014).

Joint attention is a key concept of social cognition (e.g., Carpenter et al., 1998; Tomasello et al., 2005). In order to communicate, actor and addressee must coordinate their attention so that they are jointly focused on the same referent. This is not a trivial task because it presupposes that the participants in a communicative act conceive of each other as mental or intentional agents who look at the world from different perspectives. The ability to understand another person’s perspective is a basic capacity of the human mind that evolves only gradually in preschool children and is much less developed in other species (Tomasello, 1999). Most research on joint attention has been concerned with nonverbal means of communication, notably with pointing and eye gaze (e.g., Carpenter et al., 1998; Liszkowski et al., 2006); but, of course, joint attention can also be coordinated by linguistic means. In particular, demonstratives serve to create and to manipulate joint attention in face-to-face communication (Clark, 1996; Diessel, 2006).

The term “deixis” is used in different ways by different scholars (see Fricke, 2014 for discussion). Following Bühler (1934), many researchers apply the term to linguistic expressions that are semantically contingent on a particular point of reference, which Bühler called the “origo” (Bühler, 1934: 117). The origo is the center of a coordinate system, i.e., a deictic frame of reference, which, in the unmarked case, is grounded by a speaker’s body, but which can be shifted to another person and construed in flexible ways (cf. Diessel, 2014; Stukenbrock, 2015).

Bühler’s work has been very influential (cf. Diessel, 2012a; Fricke, 2014), but in some of the recent literature, the term deixis is used in a more general way than proposed by Bühler. According to Levinson (2004), deictic expressions are linguistic elements with “built-in-contextual parameters” that must be specified by the context to be fully understood. While the speaker’s body may provide contextual cues for the interpretation of demonstratives in a particular speech situation, Levinson and others have explicitly argued against an egocentric, body-oriented concept of deixis (e.g., Levinson, 2003: 71; Peeters and Özyürek, 2016).

The different views of deixis are key to understanding why there is so much disagreement about the nature of demonstratives in the current literature. As we will see, while some researchers conceive of demonstratives as a particular class of spatial terms that are ultimately based on our bodily experience with concrete objects in space, other researchers argue that demonstratives are primarily used for social and interactive purposes and that space and embodiment are much less important to the study of deixis than commonly assumed.

This paper provides a critical review of current linguistic and psycholinguistic research on demonstratives. We begin with research on demonstratives in linguistic typology, historical linguistics, language acquisition, and signed language, and then turn to the debate about the use of demonstratives in spatial language and social interaction. To preview our conclusion, we will argue that an egocentric, body-centered view of deixis is perfectly compatible with the view that demonstratives are used for both spatial and interactive purposes.

### Universality

Demonstratives have a number of important properties that characterize them as a very special class of linguistic expressions (Diessel, 2006). To begin with, demonstratives are likely to be universal. Recent research in typology has argued that languages are much more diverse than commonly assumed in theoretical linguistics and cognitive science. According to Evans and Levinson(2009: 2), “languages differ so fundamentally from one another at every level of description (sound, grammar, lexicon, meaning) that it is very hard to find any single structural property they share.”

Yet, while language universals are rare and difficult to find, they DO exist. One aspect all languages seem to share is a particular class of demonstratives. Although Evans and Levinson do not mention demonstratives in their programmatic paper on the “myth of language universals,” there have been several large-scale typological studies suggesting that demonstratives are very likely to exist in all languages (e.g., Himmelmann, 1997; Diessel, 1999; Dixon, 2003; Breunesse, 2019; see also Levinson, 2018). The universality of demonstratives stands in sharp contrast to the cross-linguistic distribution of other closed-class items. As Evans and Levinson (and others) have noted, many languages lack adpositions, determiners, auxiliaries, conjunctions, case markers, copulas and third person pronouns. Yet, demonstratives seem to be universal.

Note that this does not concern the word class functions of demonstratives. Above we have mentioned the distinction between nominal demonstratives (e.g., *this/that*) and adverbial demonstratives (e.g., *here/there*), which concerns the analysis of demonstrative word classes. Like English, many other languages distinguish between nominal demonstratives functioning as pronouns or determiners and adverbial demonstratives functioning as spatial adverbs (Dixon, 2003). Yet, while this distinction is common, it is NOT universal. Acehnese, for instance, has three deictic particles, *nyoe, nyan* an *jêh*, glossed by (1985: 130) as “this,” “that, close” and “that, far,” respectively, that can be used as pronouns (cf. 1a) or spatial adverbs (cf. 1b). However, there are no language internal criteria that would justify a categorical division between nominal and adverbial demonstratives in Acehnese (Durie, 1985: 130–4).

(1) Acehnese (Austronesian, Indonesia)
  a. *bek    neu = peugah*   ***nyan***   *bak = lôn*
    don’t   2 = tell      that   to = I “Don’t tell me that.” (Durie, 1985: 49)
  b. ***nyoe***   *na   peng*
    here      be   money
    “Here   is my money.” (Durie, 1985: 132).

Moreover, the word class categories of demonstratives do not only comprise pronouns, determiners and spatial adverbs. If we look at demonstratives from a cross-linguistic perspective, we also find manner demonstrative adverbs (König, 2012), demonstrative identifiers (Diessel, 1999), demonstrative presentatives (Treis, 2018) and demonstrative verbs (Guérin, 2015). Mauwake, for example, has demonstrative verbs that occur with tense and verbal agreement affixes, as in example (2). Demonstrative verbs are rare, but have also been found in various other languages including Dyirbal (Dixon, 2003), Mapuche (Smeets, 1989), Komnzo (Döhler, 2018), Yukaghir (Maslova, 2003) and Quechua (Shimelman, 2017) (see Guérin, 2015 for a cross-linguistic overview).

(2) Mauwake (Trans New Guinea, Papua New Guinea)
  *nomokowa*     *unowa*      ***fan-e-mik***,*…*
    2SG/PL.brother   many     here-PST-1/3PL
    “Many of your brothers are here, ….” (Berghäll, 2015: 266).

In general, some languages use a single series of demonstratives across a wide range of contexts, but other languages have elaborate systems of demonstrative word classes (cf. Table 1). Yet, while the word class categories of demonstratives are language- and construction-particular, typologists agree that all languages have a special class of demonstratives.

**TABLE 1:** Examples of demonstrative word class systems.

		DET	PRO	ADV.SPACE	ADV.MANNER	IDENTIFIER	VERB
French	PROX	*celui/celle-(ci)*	*ce/cette-(ci)*	*ici*	*ainsi*	*ce*	*(voici)*
	DIST	*celui/celle-(là)*	*ce/cette-(là)*	*lá*			*(voilà)*
Mauwake	PROX	*fain*		*fan*	*feenap*		*fan-PST-AGR*
	DIST	*nain*		*nan*	*naap*		*nan-PST-AGR*
Acehnese	PROX	*nyoe*
	MED	*nyan*
	DIST	*jêh*	

**FIGURE 1:**
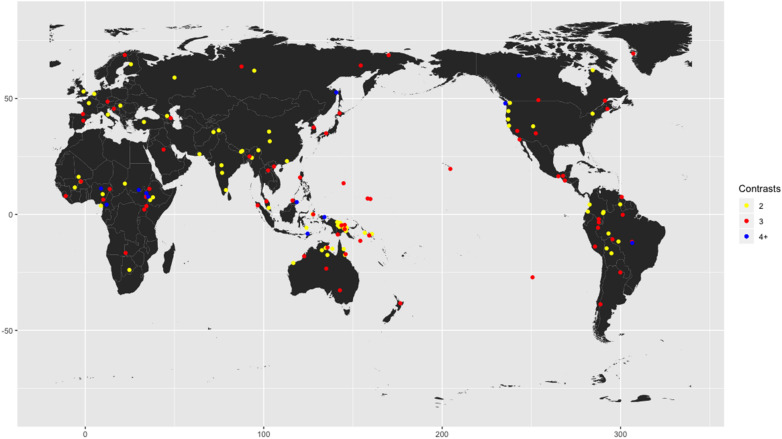
Number of distance contrasts in spatial demonstrative adverbs in a 150 language sample.

What is more, not only the existence of demonstratives is likely to be universal, but also the distinction between proximal and distal terms may be a universal property of language (Diessel, 1999; Dixon, 2003; Breunesse, 2019). Some languages have “neutral demonstratives” that are not deictically contrastive. The French demonstrative *ça*, for instance, is distance-neutral. Recent research suggests that neutral demonstratives are cross-linguistically more common than previously assumed in the typological literature (Levinson, 2018). Nevertheless, while demonstratives are not generally marked for distance, the available data suggest that all languages have at least two distance-marked demonstratives that correspond to English *here* and *there*. Figure 1 shows the number of distance contrasts in spatial demonstrative adverbs in a representative sample of 150 languages (the language sample is described in Supplementary Datasheet 1 in the Supplementary Materials).

As can be seen, the majority of languages in this sample have two or three distance terms (two-term: *N* = 72; three-term: *N* = 66). Larger systems with four or more terms are rare (*N* = 12); but note that some languages have spatial demonstrative adverbs indicating elevation, direction or visibility (not shown in Figure 1), in addition to distance (cf. Forker, 2019, this volume).

### Language Evolution

Another aspect that characterizes demonstratives as a special class is their role in language change and language evolution. Both linguists and cognitive scientists have often argued that language has evolved from gesture (e.g., Arbib, 2012; Liszkowski et al., 2012). The hypothesis is intriguing, but difficult to evaluate. Since there are no historical records of early human communication, it is impossible to study the evolution of gesture and speech directly. Nevertheless, there is good evidence from diachronic linguistics that demonstratives, which are commonly accompanied by deictic gestures (see below), have emerged early in language evolution (Diessel, 2013).

In the historical literature, it is often assumed that all grammatical function morphemes are ultimately based on content words, notably on nouns and verbs (Bybee, 2003; Hopper and Traugott, 2003). Yet, several studies have pointed out that although demonstratives are closed-class items, they are not etymologically related to nouns and verbs (Himmelmann, 1997: 20; Dixon, 2003). In particular, Diessel(1999, 2006, 2013, 2014) has argued that the diachronic origins of demonstratives are unknown. Considering data from several hundred languages, Diessel did not find a single language in which demonstratives are derived from content words, suggesting that demonstratives are fundamentally distinct from other closed-class items.

Heine and Kuteva (2007) have challenged this claim, arguing that demonstratives have evolved from motion verbs (see also Frajzyngier, 1996: 159; Heine and Kuteva, 2002: 146). The main piece of evidence for this hypothesis comes from a few African languages, in particular from !Xun, in which a verb meaning “go” is phonetically similar to a distal demonstrative. There are no historical records to investigate the proposed development in these languages. However, since motion entails distance, Heine and Kuteva(2007: 76–7) maintain that their analysis is not only suggested by the phonetic overlap between the verb “go” and the distal demonstrative “that,” but also by semantic factors.

We are not convinced by this analysis. To the best of our knowledge, there is no widespread phonetic similarity between demonstratives and motion verbs and the conceptual link between motion and distance is not sufficient to postulate a general grammaticalization path from “go” to “that.” Moreover, even if it turns out that demonstratives and motion verbs are diachronically related in some languages, the direction of the relationship could be the other way around. Heine and Kuteva assume that motion implies distance, but it is equally plausible that the indication of a distant referent is interpreted as a request for movement.

As it stands, we are not aware of any language for which we can be certain that demonstratives have evolved from motion verbs. What we do find in some languages are demonstratives that have coalesced with verbs (Brugmann, 1904; Evans, 1990; Vindenes, 2017). In French, for example, the deictic presentatives *voici* and *voilà* are historically derived from the singular imperative form of the verb *voir* “see/look” and the spatial demonstratives *ici* “here” and *là* “there.” In other languages, demonstratives have merged with copulas (e.g., in Komnzo; see Döhler, 2018: 126-7) or posture verbs (e.g., in Gunwingguan; see Evans, 1990). However, while these developments explain why the demonstratives of some languages include a verb root, or why they are inflected for tense, they do not explain where demonstratives come from.

In general, although there is no apriori reason to exclude the possibility that a demonstrative may evolve from a motion verb, the available data suggest that, if this has ever happened, it is a rare phenomenon that does not explain the diachronic origins of demonstratives as a cross-linguistic class (Diessel, 2006, 2013).

**FIGURE 2:**
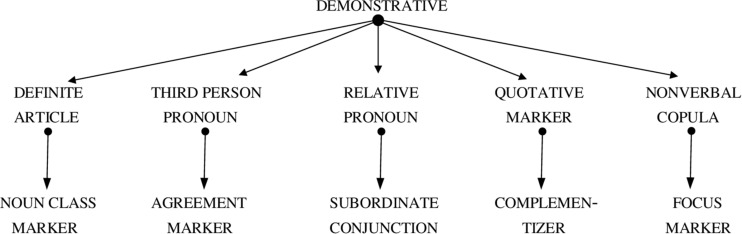
The grammaticalization of demonstratives: Some frequent cross-linquistic paths.

What is more, demonstratives are not only old and non-derived, they also play a key role in the diachronic evolution of grammar. Research on grammaticalization has been mainly concerned with the development of function morphemes from nouns and verbs. There is plenty of evidence that adpositions, auxiliaries, case markers and many other types of grammatical morphemes have evolved from content words. Yet, what is often overlooked, or not sufficiently explained in the grammaticalization literature, is that demonstratives provide a second major source for grammatical function morphemes (Himmelmann, 1997: 115–155, Diessel, 1999, 2019: 167–171; Diessel and Breunesse, 2020). Across languages, demonstratives are commonly reanalyzed as definite articles, third person pronouns, relative pronouns, quotative markers and nonverbal copulas, which in turn often develop into noun class markers, agreement affixes, subordinate conjunctions, complementizers and focus markers (see Figure 2). While some of these morphemes may also arise from content words, there can be no doubt that demonstratives are of fundamental significance to the diachronic evolution of grammar, as already suggested in the classic works of Brugmann (1904) and Bühler (1934).

### Language Acquisition

There is little research on the acquisition of demonstratives, but in a classic paper, Clark (1978) made three important claims. First, she argued that the acquisition of verbal deixis builds on children’s prior use of deictic gesture; second, she claimed that demonstratives are usually among children’s first words; and third, she argued that demonstratives are very frequent in early child language:

“Among the earliest words acquired is usually at least one deictic word—a word invariably used together with a deictic gesture. A deictic word based on *there* or *that* … often appears in the first ten words of English-speaking children, certainly within the first 50 words” (Clark, 1978: 95).

These claims are widely cited in the literature, but to date only few studies have examined the acquisition of demonstratives in development (empirically). Several studies show that the development of demonstratives seems to be quite protracted, with adult-like uses emerging long after children start producing demonstratives (de Villiers and Villiers, 1974; Webb and Abrahamson, 1976; Clark and Sengul, 1978; Tanz, 1980; Küntay and Özyürek, 2006). However, the only study we know that is specifically concerned with the relationship between pointing and demonstratives in young children is a recent paper by Todisco et al. (2020).

Using data from reading sessions in which participants referred to animals in a picture book, Todisco et al. (2020) analyzed the interaction between verbal and nonverbal means of deictic reference in Italian-speaking children aged 20 to 31 months. While these children are (already) too old to examine the transition from gesture to speech, Todisco et al. observed that young children frequently combine demonstratives and pointing gesture, and do so in a synchronous manner, with the peak of the pointing gesture produced at the same time as the deictic vocalization. Moreover, joint attention on an object (both infant and caregiver looking at the intended referent) was found to immediately precede deictic communication in the vast majority of deictic episodes.

The main piece of evidence for Clark’s hypothesis that demonstratives are among children’s first words comes from diary and observational research (Nelson, 1973; Bates, 1976). Specifically, these studies report that children’s early pointing gestures are frequently accompanied by vocalizations such as [e], [a?], or [da] that may be seen as precursors of demonstratives (Clark, 1978: 95). If this is correct, demonstratives are usually among the earliest words children produce.

Caselli et al. (1995) presented data that raised doubts about this claim. Analyzing parent reports of young English and Italian children, compiled with the MacArthur-Bates Communicative Development Inventory (CDI), these researchers did not find any demonstratives among children’s first 50 words. However, since parent reports may not provide a reliable measure for the appearance of closed-class function words (Salerni et al., 2007), we decided to look at children’s early demonstratives in naturally occurring child speech.

Using data from the CHILDES database (MacWhinney, 2000), we investigated records of spontaneous speech from 20 children, learning four different languages: English (*N* = 10), Dutch (*N* = 3), Hebrew (*N* = 4), and Japanese (*N* = 3). We selected these children based on two criteria: age and the amount of data available for each child. All 20 children were younger than 25 months and their data include a minimum of 3400 words per child. The results of this study are summarized in Supplementary Datasheet 2 of the Supplementary Materials.

Overall, the data comprise 206,188 child words. Since most of these words were produced by children beyond the one-word stage, the data are not fully appropriate to examine the appearance of children’s very first words. Nevertheless, while one would need other types of data to determine the precise age when children begin to use demonstratives, the data strongly suggest that demonstratives are generally among children’s early words. As it turns out, there was at least one demonstrative in the first file of all 20 children regardless of their age. Even the youngest children, aged 10 to 17 months (Laura, Naomi, Judith, Peter, Meinder, Smadar, Lior, Nanami, Asoto, Kiichan), used demonstratives from early on.

The vast majority of children’s early demonstratives occur in one-word utterances, or less frequently, together with a noun. They are embedded in parent-child interactions in which the participants seek to focus the other person’s attention onto a referent in the surrounding situation. Here are some typical examples from the English data:

(3) Naomi (14 months)^∗^CHI: Kit(ty) kit(ty).
  ^∗^MOT: Okay, are you done with looking at the pictures?
  ^∗^MOT: Are you going to give them to me?
  ^∗^CHI: **Dere** [ = there].
  ^∗^MOT: Okay you can give them to me.

(4) Laura (18 months)^∗^CHI: Matthoo [ = Matthew].
  ^∗^MOT: And Matthew.
  ^∗^MOT: Where are they?
  ^∗^CHI: **There**.
  ^∗^MOT: There?

(5) Eve (18 months)^∗^MOT: I don’t think so.
  ^∗^MOT: Mr. Fraser has coffee.
  ^∗^MOT: Mr. Fraser’s drinking coffee.
  ^∗^CHI: **That**.
  ^∗^MOT: What is that?

(6) Eve (18 months)^∗^MOT: The ducks say what?
  ^∗^CHI: **That**.
  ^∗^MOT: What is that?
  ^∗^CHI: **That** radio.
  ^∗^MOT: What?

(7) Eve (18 months)^∗^ADL: That’s very good.
  ^∗^CHI: I did it.
  ^∗^CHI: **There**.
  ^∗^CHI: **There** Fraser.
  ^∗^ADL: That’s a nice box of books.

While the CHILDES transcripts do not provide (systematic) information about the context and use of gesture, it is clear from the data that children’s early demonstratives refer to objects and locations in their vicinity and that many of these early uses are accompanied by gesture (as indicated on the “action tier”), consistent with the findings of Todisco et al. (2020). The data also show that demonstratives are very frequent in early child language. As can be seen in Table 2, demonstratives account for a very large proportion of children’s early words, ranging from a mean of 5.9% in Dutch to a mean of 8.3% in English.

**TABLE 2:** Raw frequencies and mean proportions of demonstratives in early child speech.

	Number of children	Age range	Corpus size (child)	DEM total	Mean proportions
English	10	1.02–2.0	103329	8478	8.27%
Dutch	3	0.10–2.0	20991	869	5.88%
Hebrew	4	1.04–2.0	34852	3101	7.76%
Japanese	3	1.00–2.0	47016	3277	7.82%
Total	20		206188	15725	

Moreover, if we look at the frequencies of individual words, we find a demonstrative at the top of the word frequency lists of 8 of the 20 children. Apart from demonstratives, children made extensive use of pronouns (e.g., *it*), determiners (e.g., *the*) and interjections (e.g., *oh, yeah, no*); but with the exception of “mummy” and some proper names, there were hardly any nouns (or verbs) among the 20 most frequent words at this age, suggesting that demonstratives are the preferred means of linguistic reference in early child language.

Comparing children across the four languages, we found a conspicuous asymmetry in the use of proximal and distal terms. The English- and Dutch-speaking children used more distal demonstratives than proximal demonstratives (English: 26.2% proximal vs. 73.8% distal; Dutch: 30.3% proximal vs. 69.7% distal), but the Hebrew- and Japanese-speaking children used primarily proximal terms (Hebrew: 97.1% proximal; Japanese: 90.1% proximal). There were also some medial demonstratives in the Japanese data (3.9% medial), but distal demonstratives were rare in both Hebrew (2.8% distal) and Japanese (5.2% distal). Since children’s mothers used very similar proportions of proximal and distal terms, it seems reasonable to assume that children tend to use demonstratives that are frequent in the ambient language.

More research is needed to investigate the acquisition of demonstratives and the alternation between proximal and distal terms in early child language. However, while our data are not sufficient to verify Clark’s claim that demonstratives are always among children’s first 50 words, they strongly suggest that children begin to produce demonstratives early and that demonstratives are among the most frequent words in early child language.

### Multimodality

One of the most conspicuous properties of demonstratives is that they are frequently accompanied by nonverbal means of deictic reference, notably by pointing and eye gaze (Enfield, 2003; Stukenbrock, 2015; Levinson, 2018). The multimodal use of demonstratives has been investigated from different perspectives with a variety of methods.

First, linguistic field workers have developed particular elicitation tools to examine the interaction between demonstratives, pointing and gaze in different contexts. Of particular importance is the questionnaire developed by Wilkins (2018), which has been used in a large number of studies on languages across the world (see the recent collection of articles in Levinson et al., 2018). While the Wilkins questionnaire is not specifically designed to probe into multimodal communication, this research strongly suggests that the combination of demonstratives with pointing and gaze is cross-linguistically very common.

However, while multimodality may be a universal trait of demonstrative reference, there are interesting differences in the way demonstratives are combined with nonverbal strategies of deixis. For instance, while it is by no means uncommon for speakers of English to use (exophoric) demonstratives without a co-occurring gesture, reports of linguistic field workers suggest that there are languages in which certain types of demonstratives are generally accompanied by pointing or gaze. In Yélî Dnye, for example, proximal demonstratives seem to require a pointing gesture, or at least gaze, unless the referent is being manipulated (Levinson, 2018: 32). Other languages in which pointing or gaze appear to be “obligatory” with certain demonstratives include Goemai (Hellwig, 2003: 263), Kilivila (Senft, 2004: 62), Yucatec (Bohnemeyer, 2018), Warao (Herrmann, 2018), and Tiriyó (Meira, 2018). Interestingly, in many of these languages it is the proximal demonstrative that is tied to gesture (Levinson, 2018: 32-3).

Second, the multimodal use of demonstratives has been examined with methods of conversational analysis (e.g., Laury, 1997; Hindmarsh and Heath, 2000; Strauss, 2002; Enfield, 2003; Eriksson, 2008; Etelämäki, 2009; Jarbou, 2010; Stukenbrock, 2015; Gipper, 2017). Using video recordings of naturally occurring speech, these studies provide in-depth analyses of multimodal demonstratives in different contexts. One aspect that is emphasized in this research is that demonstratives are primarily used for interactive purposes rather than for spatial reference (see below). Another finding is that demonstratives are not only combined with prototypical pointing gestures, involving the extended arm and index finger, but also with various forms of “bodily displays” including touching, reaching, holding and picking up (Eriksson, 2008; see also Hindmarsh and Heath, 2000; Enfield, 2003; Talmy, 2018).

Finally, there are a number of psycholinguistic studies that have examined the multimodal use of demonstratives with experimental methods (e.g., Bangerter, 2004; Piwek et al., 2008; Lücking et al., 2015; Cooperrider, 2016; Garciá et al., 2017). Most of this research is concerned with demonstratives and pointing, but Garciá et al. (2017) looked at the interaction between demonstratives and gaze (see also Todisco et al., 2020 and Stukenbrock, this volume). Using an experiment in which participants had to instruct another person to move an object on a tablet, they tested the effect of eye gaze on spatial language under two conditions: the gaze condition, in which participants could see each other’s eyes, and the no-gaze condition in which their eyes were hidden behind goggles. As expected, in the gaze condition, participants made extensive use of demonstratives, but in the no-gaze condition, they resorted to other, non-deictic means of spatial language, suggesting that speakers shun away from verbal deixis when gaze is not available as a communicative device.

In accordance with this finding, Bangerter (2004) observed that the availability of gesture has a significant impact on speakers’ use of demonstratives. When gesture is available, speakers prefer short deictic descriptions; but when gesture is not available, they tend to use longer nondeictic descriptions. In addition, Bangerter found that the combination of demonstrative and gesture varies with distance. Other things being equal, the gestural use of demonstratives is much more frequent for nearby referents than for referents far away (see also Piwek et al., 2008). Since far-away referents are often difficult to identify by gesture (Lücking et al., 2015), one might hypothesize that the correlation between distance and pointing is ultimately motivated by the ambiguity of distant pointing. Good evidence for this hypothesis comes from a study by Cooperrider (2016), who found that speakers extend the gestural use of demonstratives to far-away referents if they are given a laser pointer, making it possible to identify a distant referent that cannot be unambiguously identified by manual gesture.

### Signed Language

Signed language abounds with pointing gestures, but the status of pointing signs is controversial (e.g., Liddell, 2000; Cormier et al., 2013; Johnston, 2013; Fenlon et al., 2019). Like demonstratives, pointing signs can target either a perceptually accessible referent in the surrounding situation or a discourse referent in signed space. In the latter case, an absent referent is located on a horizontal plane in front of the signer, where it is available as a “locus” for subsequent reference through pointing.

Following Friedman (1975) and others, it has long been assumed that pointing gestures have the status of lexical signs in signed language. They are commonly analyzed as particular types of words functioning as pronouns, determiners, adverbs and other word classes (Friedman, 1975; Klima and Bellugi, 1979). However, some of the more recent literature has questioned this view, arguing that pointing signs are distinct from lexical signs (Liddell, 2000; Cormier et al., 2013; Johnston, 2013).

All researchers agree that pointing is of central significance to reference in signed language, but given that pointing is also commonly used as co-speech of spoken language, it is not immediately clear why pointing signs should be regarded as words rather than as genuine gestures (Engberg-Pedersen, 2003). There is an ongoing debate about this issue in the current literature.

Some researchers claim that pointing signs are fundamentally distinct from pronouns or determiners (Liddell, 2000) and emphasize that most of the features that characterize pointing signs in signed language are also characteristic of deictic points in co-speech (Liddell, 2000; see also Engberg-Pedersen, 2003; Johnston, 2013). Other researchers argue that, while pointing signs are (superficially) similar to pointing in spoken language, they are more constrained in meaning and form than ordinary pointing gestures. For instance, in one study, Fenlon et al. (2019) compared video data of 24 signers of British signed language to video data of 27 speakers of American English and found that, on balance, signers’ pointing signs were more reduced, more consistent, and more integrated with other aspects of the linguistic system than the pointing gestures of speakers’ co-speech.

As it stands, the issue is unresolved (see Cormier et al., 2013 for discussion); but irrespective of the view a particular researcher holds in this debate, there is widespread consensus that pointing signs have a particular status in signed language: they are “semi-conventionalized” (Johnston, 2013) and combine aspects of “word and gesture within a single form” (Meier and Lillo-Martin, 2010: 356; see also Cormier et al., 2013).

What is more, some recent studies explain the particular status of pointing signs in signed language by grammaticalization (Pfau and Steinbach, 2011; Fenlon et al., 2019). More precisely, Pfau and Steinbach (2011) hypothesized that (many) pointing signs can be seen as grammaticalized pointing gestures that have evolved along a grammaticalization path leading from genuine pointing gestures via locative pointing signs to determiners, personal pronouns and agreement markers. Since there are almost no diachronic data of signed languages, the hypothesis is difficult to verify (Pfau and Steinbach, 2011: 384). However, interestingly, Coppola and Senghas (2010) present data from Nicaraguan sign language that could be interpreted as evidence for the proposed grammaticalization path.

Nicaraguan sign language emerged as a new language in the late 1970s when deaf children were brought together for the first time at school. Earlier, deaf people had very little contact with each other and signed only at home to communicate with hearing people around them. Comparing pointing signs of four “homesigners” with pointing signs of different cohorts of signers who used Nicaraguan sign language at school (starting at different stages of the emerging language), Coppola and Senghas found that homesigners’ pointing signs were almost exclusively used to indicate a place, whereas the pointing signs of the three cohorts who used Nicaraguan sign language at school also included abstract points functioning as determiners and personal pronouns which seem to have evolved from locative points by grammaticalization.

Finally, while pointing signs share many properties with demonstratives in spoken language, it is unclear if the deictic points of signed language are marked for distance, like most demonstratives in spoken language, or if they are distance-neutral, like the deictic points of co-speech. The only study we know that has been explicitly concerned with the expression of distance in signed language is Morford et al. (2019). Using an experimental paradigm in which bilingual signers of American sign language had to coordinate their actions in a cooperative task, these researchers observed that points to distal referents were often accompanied by “facial compressions” such as eye squinting, head tilt and cheek raising, which only rarely appeared with points to proximal referents. However, the same facial compressions also occurred when the addressee had misunderstood a previous referent. In addition, Morford et al. observed that pointing signs of distal referents were more often used with a straight index handshape and an arc trajectory than pointing signs of proximal referents, but this was not statistically significant. Only future research can show if the distinction between proximal and distal deictics also occurs in signed language or if the pointing signs of signed language are distance-neutral (possibly because distance is an emergent property of deictic pointing that has not yet been grammaticalized in signed languages, which tend to be much younger than spoken languages).

### Perceptual Space

We now turn to the above-mentioned debate about the nature of deixis and demonstrative reference. Recall that there are two different views of deixis. Some researchers conceive of (spatial) deixis as an egocentric, body-oriented strategy to provide orientation in space (e.g., Bühler, 1934; Coventry et al., 2008; Diessel, 2014); but other researchers dispute the pivotal role of speakers’ body for the study of deixis and argue that demonstratives are primarily used for social and interactive purposes rather than for spatial reference (e.g., Jarbou, 2010; Peeters and Özyürek, 2016; Gipper, 2017).

In what follows, we discuss studies from both sides of the debate. We begin with research supporting the egocentric, body-oriented view of spatial deixis and then turn to research that has emphasized the social and interactive functions of demonstratives.

At the heart of the current debate about demonstrative reference is the alternation between proximal and distal terms. Traditionally, this alternation is explained by the relative distance between referent and origo. Crucially, “relative distance” must not be confused with “physical distance.” As Bühler (and many others) have pointed out, speakers’ choice between proximal and distal demonstratives is contingent on language users’ conceptualization of the speech situation rather than on physical properties of space. Consider, for instance, the following examples of the proximal demonstrative *here* (cf. Diessel, 2012b: 2410).

(3) a. Here on my legb. Here in this room
  c. Here in London
  d. Here in Europe
  c. Here on this planet.

What these examples show is that the region included in the origo varies with the construal of the speech situation. In example (3a), *here* refers to a small spot on speaker’s leg, but in all other examples, it refers to a much larger region, which generally includes the speaker but may also include the addressee. The distal term *there* is used in contrast to *here*. It can refer to any location as long as the location is not included in the region conceptualized as the origo. Thus, while *here* and *there* refer to locations in different distance to the speaker, the distance features of these terms are determined by interlocutors’ conceptualization of the speech situation rather than by objective properties of metrical space (e.g., Bühler, 1934; Enfield, 2003).

However, while the alternation between proximal and distal demonstratives cannot be defined in terms of physical space, a number of recent studies have argued that the encoding of distance in demonstratives is ultimately based on our bodily interaction with concrete objects in (real) space. These studies draw on research in neuropsychology indicating that objects in peripersonal space are processed in fundamentally different ways from objects in extrapersonal space (e.g., Goodale and Milner, 1992). Peripersonal space is the region of space in which a person can interact with objects and animate beings, by reaching, grasping or touching. Extrapersonal space, in contrast, is the region of space in which objects and animate beings are only perceptually accessible but not available for (physical) interaction (see Bufacchi and Iannetti, 2018 for a review).

Considering this distinction, Coventry and colleagues hypothesized that the universal contrast between proximal and distal demonstratives has its roots in the neuropsychological distinction between peripersonal and extrapersonal space (Coventry et al., 2008, 2014; Gudde et al., 2016; see also Rocca et al., 2019b). This hypothesis was first proposed by Kemmerer (1999), who then dismissed it, mainly because proximal demonstratives are not only used for reachable objects.

However, while the alternation between proximal and distal demonstratives is very flexible, there is good reason to assume that demonstrative choice is ultimately grounded in the vision and action systems, i.e., in the distinction between peripersonal and extrapersonal space. In early research on this topic, peripersonal space was primarily defined in terms of the actor’s body, notably the actor’s perimeter of arm’s reach (Kemmerer, 1999); but more recent research suggests that the distinction between peripersonal and extrapersonal space is mainly determined by the way a person interacts with objects rather than by concrete body parts (see Bufacchi and Iannetti, 2018 for a recent discussion of relevant findings).

**FIGURE 3:**
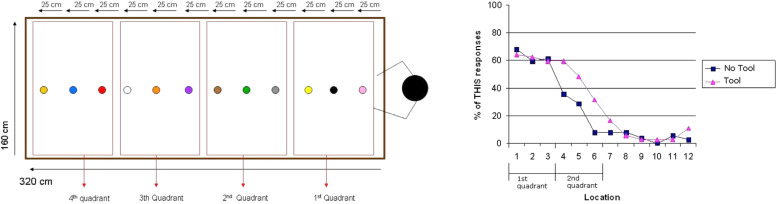
Experimental set-up used by Coventry et al. (2008) (left panel) and associated results (right panel).

Building on these considerations, Coventry et al. (2008) conducted a series of experiments with speakers of English and Spanish in order to investigate the potential influence of peripersonal space on speakers’ choice of a particular demonstrative. Using a new experimental paradigm in which participants could choose between proximal and distal terms in order to refer to objects at different distances from the speaker (Figure 3 left panel), they found a strong preference for proximal demonstratives if speakers could reach the referent. Crucially, while *this* was usually confined to objects in arm’s reach, Coventry et al. showed that speakers extend the use of proximal demonstratives to referents at a greater distance if they can use a tool, e.g., a stick, in order to reach it (Figure 3 right panel), indicating that it is not speakers’ body *per se* but the (in)ability to interact with an object that affects their choice of a particular deictic term.

The results were replicated in several follow-up studies with speakers of other languages under somewhat different experimental conditions (cf. Coventry et al., 2014; Gudde et al., 2016; Caldano and Coventry, 2019; Reile et al., 2020). Other evidence supporting a distance-based analysis of demonstrative reference comes from an EEG experiment by Stevens and Zhang (2013) and a behavioral study by Bonfiglioli et al. (2009).

Taken together, this research provides compelling evidence for an egocentric, body-oriented view of spatial deixis in which speakers’ choice of a demonstrative is (often) motivated by the possibility of interaction between referent and origo, which ordinarily correlates with distance. However, in addition to relative distance, there are various other factors that can influence demonstrative choice (cf. Coventry et al., 2014).

### Interactional Space

If one were to adopt a purely distance-based account, one important aspect “missing” is the role of the hearer (Jungbluth, 2003). Accounts of the demonstrative systems with three or more terms often consider whether such systems are “distance-based” (e.g., with a medial distance term) or whether they might be “person-centered.” For example, Spanish has three demonstratives, *este, ese* and *aquel*, that are often described in grammars as being parallel to the distinction between first, second and third person. On this view, *este* refers to an object near the speaker, *ese* indicates a referent near the hearer, and *aquel* specifies a referent far away from both speech participants (Anderson and Keenan, 1985). In contrast, Anderson and Keenan (1985) consider the Japanese three-term demonstrative system a distance-based system, with the terms *kore*, *sore* and *are* representing increasing distance from the speaker (but see Hasegawa, 2012).

The distinction between person-oriented and distance-oriented systems has been prominent in cross-linguistic research on demonstratives (Anderson and Keenan, 1985; Diessel, 1999; Dixon, 2003). Yet, recent research suggests that this distinction is note quite appropriate to characterize demonstratives in three- and four-term systems (cf. Levinson, 2018). One reason for this is that the position of the hearer can influence the conceptualization of space in several different ways. As Jungbluth (2005) has demonstrated, based on data from Spanish, the influence of hearers’ position on demonstrative choice varies with the constellation of speaker and addressee in a particular situation (see also Jungbluth, 2003).

**FIGURE 4:**
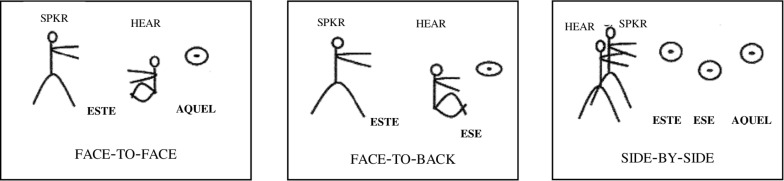
Constellation of speech participants in Jungbluth’s study of Spanish demonstratives (adopted from Jungbluth, 2003).

Using an elicitation task, Jungbluth examined the use of *este, ese* and *aquel* in three basic constellations: face-to-face, face-to-back and side-by-side. In face-to-face conversation, every object included in interlocutors’ shared field of vision was referred to by *este*, even if the referent was close to the addressee; yet, referents outside of interlocutors’ shared space were referred to by *aquel* (Figure 4 left panel). In face-to-back conversation, *ese* referred to objects in hearers’ immediate field of vision, whereas *este* was preferred for referents near the speaker, which the hearer could not see (Figure 4 middle panel). Finally, in side-by-side conversation, *este, ese* and *aquel* were used to differentiate between three different referents on a relative distance scale (Figure 4 right panel).

Thus, while there are situations in which *ese* indicates a referent near the hearer, Jungbluth maintained that Spanish does not have a simple hearer-oriented system as commonly assumed in the typological literature. Rather, the use of all three Spanish demonstratives varies with the constellation of the speech participants and the location of the intended referent (see also Coventry et al., 2008). Generalizing across the constellations shown in Figure 4, Jungbluth argued that the main determinant for speakers’ choice of a particular demonstrative in Spanish is the “conversational dyad” or “shared conversational space.”

Similar analyses have been proposed by other scholars for other languages with both two- and three-term systems (Hanks, 1990; Burenhult, 2003; Enfield, 2003; Piwek et al., 2008; Peeters et al., 2015). For instance, Peeters et al. (2015) have argued, based on data from EEG experiments, that the “construal of shared space” determines the alternation between proximal and distal demonstratives in Dutch. The results of this study are complex, but Peeters et al. interpret N400 effects as evidence that proximal demonstratives are preferred in face-to-face constellations for referents in shared space (but only if there is no alternative referent outside of the conversational dyad). Since these effects occurred regardless of the relative distance between speaker and referent, Peeters et al. claim that their results are not consistent with an egocentric and distance-based account of spatial deixis (see also Peeters and Özyürek, 2016).

In a similar vein, Piwek et al. (2008) argue that cognitive accessibility, rather than distance in space, motivates speakers’ choice of a particular demonstrative in Dutch. Using a dialogue game in which participants instructed an experimental collaborator to build a lego model, they found that distal demonstratives are preferred for highly accessible referents, whereas low-accessible referents are commonly referred to by proximal demonstratives (see also Kirsner, 1979).

In general, there is a large body of research indicating that demonstrative choice is influenced by shared space and accessibility (Ariel, 1990; Gundel et al., 1993; Laury, 1997; Burenhult, 2003; Enfield, 2003). However, while this sheds new light on demonstrative reference, it does not undermine an egocentric, body-oriented account of spatial deixis. On the contrary, what these studies show is that demonstratives are commonly used to coordinate interlocutors’ joint focus of attention, which typically involves body-oriented means of communication such as pointing and gaze that are produced from an egocentric perspective. Assuming that demonstratives are commonly used to manipulate joint attention in multimodal communication, we contend that demonstratives are best analyzed within an egocentric, body-oriented frame of reference.

However, crucially, while the deictic frame of reference is usually grounded in the speaker’s body (in the unmarked case), linguistic reference is never immediately determined by physical properties of the outside world—it is always contingent on the conceptualization of space (Talmy, 2000, 2018). Like all other aspects of meaning, deixis is the product of conceptual processes, such as the figure-ground organization, that influence the choice of a particular term (see also Diessel, 2019: 27-30).

What is more, while the speaker’s body is commonly interpreted as the origin of a deictic frame of reference, it must be emphasized that the origo, or deictic center, can be shifted from the speaker to another person.

### Projected Space

One of Bühler’s most important discoveries was that, while demonstratives are commonly used in perceptual space, they can also be used in spatial imagery. Bühler called this “Deixis am Phantasma” and analyzed several distinct cases (Bühler, 1934: 121-140). In the most basic case, the origo is shifted from the speaker onto another person or viewer. The phenomenon is well-known from narratives and spatial descriptions. In narrative discourse, the origo is projected from the speaker, or writer, to a protagonist or narrator who uses demonstratives with reference to objects in the story world (Ehlich, 1979). A similar phenomenon has been observed in linguistic and psycholinguistic research on spatial descriptions (e.g., Ullmer-Ehrich, 1982).

Deictic projections have been investigated in narratology and discourse analysis (Linde and Labov, 1975), but there is little (recent) research on this topic in linguistics and psychology. Stukenbrock (2014, 2015) analyzed deictic projections and other forms of Deixis am Phantasma in video recordings of conversational German. One important finding that has emerged from this research is that space deixis, time deixis and person deixis are not always aligned in spatial imagery. There are interesting blends of deictic projections in Stukenbrock’s data in which the deictic dimensions of space, time and person are disassociated from one another.

Another recent study that illustrates the importance of deictic projections for the analysis of demonstrative reference is Rocca et al. (2019c). Using a new interactional paradigm, these researchers found that participants shifted their deictic coordinate system onto a collaborator during a spatial coordination task. Considering this finding, Rocca et al. argue that speakers remap their “action space,” or “peripersonal space,” onto their “partners’ action space” in order to facilitate the collaboration between them.

Deictic projections are crucial to the current debate about the nature of demonstrative reference because they show that even in an egocentric, body-oriented theory of deixis, demonstratives are not always grounded by the speaker. If the origo is shifted, the alternation between proximal and distal terms is determined by the target of the projection rather than the speaker’s body or location. Indeed, such an approach is consistent with evidence from other spatial terms—projective adpositions (e.g., *to the left/right*; *in front of*)—where it has been shown empirically that another person’s perspective is frequently used to assign direction when speaker and hearer are misaligned (i.e., *the cup is on the (hearer’s) left*; see Tversky and Hard, 2009; Tosi et al., 2020).

### Beyond Space

Finally, like many other types of spatial expressions, demonstratives can be extended from the domain of space into non-spatial domains (cf. Bühler, 1934; Fillmore, 1997; Griffiths et al., 2019; Rocca et al., 2019a). To begin with, across languages demonstratives are commonly used with reference to time (cf. *this/that time, month, year*). There is little research on temporal demonstratives, but the extension from space to time is well-known from research on other types of expressions (Haspelmath, 1997; Boroditsky, 2002). If we think of time as a “time line,” demonstratives refer to an earlier or later point on that line.


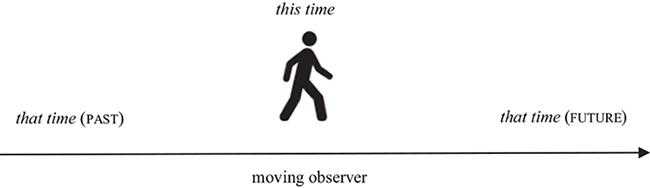


Note that the temporal use of demonstratives involves a radical reconstruction of the conceptual frame that underlies the interpretation of deixis. Spatial demonstratives are interpreted within a coordinate system that is usually evoked by speakers’ body, gaze and gesture, but can also be derived from other aspects of the context (in spatial imagery for instance) (Diessel, 2014). However, in contrast to the conceptualization of space deixis, the conceptualization of time deixis does not involve a body-oriented frame of reference, as evidenced by the fact that temporal demonstratives are not accompanied by pointing, gaze or body posture. Only one study to date has examined how demonstratives are used temporally and spatially within the same context. Griffiths et al. (2019) ran a series of experiments eliciting demonstratives to refer to objects, manipulating *where* objects were located in (virtual) space and also *when* objects appeared (e.g., objects appeared and disappeared at different times). They found that demonstratives were used according to whether the object was reachable or not, but there were no effects of time of object appearance/disappearance on demonstrative choice. One interpretation of these findings is that the spatial determinants of demonstrative use take precedence over non-spatial uses, consistent with conceptual metaphor theory (Lakoff and Johnson, 1980).

Like time deixis, discourse deixis often involves demonstratives. Since language unfolds in time, discourse is commonly conceptualized as a continuous stream of linguistic elements. There is a close connection between time deixis and discourse deixis. Both are construed as a band of successive elements that is divided into distinct areas by a moving origo. However, while the origo of time deixis is determined by the moment of speaking, the origo of discourse deixis is determined by the location of a demonstrative in the unfolding discourse.


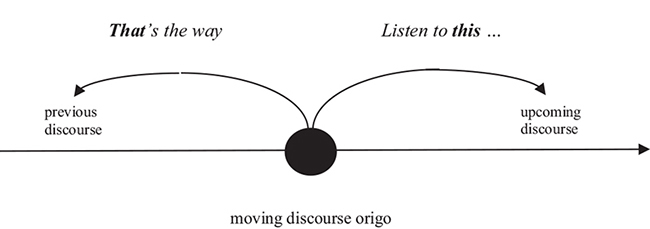


As Bühler (1934: 390) put it:

If discourse deictic expressions could speak, they “would speak more or less as follows: look ahead or back along the band of the present utterance. There something will be found that actually belongs here, where I am, so that it can be connected with what now follows. Or the other way around: what comes after me belongs there, it was only displaced from that position for relief.” [English translation from Goodwin 1990: 443]

The discourse use of demonstratives has been investigated in a large number of studies using both corpus and experimental methods (e.g., Gundel et al., 1993; Himmelmann, 1996; Kaiser and Trueswell, 2008; Kehler et al., 2008; Cornish, 2011; Fossard et al., 2012; Kehler and Rohde, 2013, 2019). The results of this research are too complex to be reviewed in this paper, but there is one finding we’d like to mention as it concerns the current debate about the nature of demonstrative reference. While the notion of relative distance is not immediately relevant to the discourse use of demonstratives, there is evidence that speakers’ choice between proximal and distal terms in discourse is influenced by the same psychological factors as demonstrative choice in perceptual space, i.e., by accessibility (Ariel, 1990), common ground (Kaiser and Trueswell, 2008) and manual affordances (Rocca et al., 2019a). In fact, Talmy (2018) argues that the discourse use of demonstratives (which he calls “anaphoric”) involves the same cognitive processes as the perceptual use of demonstratives (which he calls “deictic”). On Talmy’s account, language includes a single “targeting system” that underlies demonstrative reference in both perceptual space and discourse processing.

## Conclusion

In conclusion, in this article we have reviewed linguistic and psycholinguistic research on demonstratives from many different perspectives. There is widespread consensus in the literature that demonstratives constitute a unique class of expressions that are crucially distinct from other closed-class items: Demonstratives are likely to be universal and not derived from content words, they seem to be among the earliest and most frequent words in L1 acquisition, they are closely related to pointing, gaze and body posture, and they are of fundamental significance to the diachronic evolution of grammar.

But why are demonstratives so special? What distinguishes them from adpositions, auxiliaries and other closed-class items. We suggest that demonstratives have a particular status in language because of their communicative function to create and to manipulate joint attention (Diessel, 2006).

Joint attention is a prerequisite for social interaction, language acquisition and language evolution (Tomasello, 1999), and it is closely related to spatial deixis. While joint attention is defined as a social phenomenon, it is created by nonverbal means of deictic reference such as pointing and gaze that involve the human body, notably the actor’s body, as a source of spatial orientation. Since demonstratives are commonly combined with pointing and gaze, it is reasonable to assume that, in the unmarked case, it is the speaker’s body and gesture that provide a (deictic) frame of reference for the semantic interpretation of demonstratives.

There is compelling evidence that demonstrative reference has its roots in our bodily experience with objects in space (Coventry et al., 2008, 2014); but, crucially, deictic space must not be confused with physical space. Some recent studies have criticized the egocentric, body-oriented view of deixis because not all uses of demonstratives involve speakers’ body in physical space (e.g., Peeters and Özyürek, 2016). However, this critique is unfounded as it does not recognize the role of conceptualization in the creation of deixis. As Bühler (and many others) have noted, while the deictic center is usually grounded by a speaker’s body at the time of an utterance, it can be construed in flexible ways. As we have seen, the origo may be a small spot or a large region, it may or may not include the addressee, and it can be shifted to another person or viewer and mapped onto nonspatial domains, notably the domains of time and discourse.

It should be noted that it is often hard to compare results from studies that employ such a wide range of methodologies – from linguistic work in the field, often (by necessity) with small numbers of informants that makes generalization difficult, to controlled experimental studies with increased (statistical) power, but sampling linguistic behavior in more circumscribed situations. Nevertheless, it would seem that the factors that influence the conceptualization of a deictic frame of reference are many. Of particular importance is interlocutors’ common ground (Clark et al., 1983), but salience in sensory perception and language users’ experience with particular types of expressions are also important (Talmy, 2000). This explains why demonstrative reference is so tremendously variable. However, like many other aspects of meaning, the meaning of demonstratives is ultimately based on our bodily experience with objects in space.

## Author Contributions

The manuscript was jointly written by HD and KC. Both authors contributed to the article and approved the submitted version.

## Conflict of Interest

The authors declare that the research was conducted in the absence of any commercial or financial relationships that could be construed as a potential conflict of interest.
